# A Comprehensive Evaluation of Factors Affecting the Mental Status and Quality of Life in Breast Cancer Patients With Schizophrenia at Different Treatment Stages (Preoperative and Postoperative)

**DOI:** 10.62641/aep.v53i3.1788

**Published:** 2025-05-05

**Authors:** Manfei Qian, Liyan Wang, Huihong He, Pengbo Tao, Linyu Cai, Juying Zhang

**Affiliations:** ^1^Department of Interventional Medicine and Medical Oncology (II), Shaoxing People’s Hospital, 312000 Shaoxing, Zhejiang, China; ^2^Department of Geriatrics, Integrated Chinese and Western Medicine, Shaoxing People’s Hospital, 312000 Shaoxing, Zhejiang, China; ^3^Department of Mammary Thyroid, Shaoxing People’s Hospital, 312000 Shaoxing, Zhejiang, China; ^4^Department of Clinical Medicine City College of Zhejiang University, 310000 Hangzhou, Zhejiang, China; ^5^Department of Nursing, Hunan Institute of Traffic Engineering, 421009 Hengyang, Hunan, China

**Keywords:** breast cancer with schizophrenia, mental status, quality of life, SCL-90, WHOQOL-BREF, surgery

## Abstract

**Objective:**

Surgery, as the preferred option to extend the survival of breast cancer patients, has increasingly garnered attention for its impact on patients’ mental status and quality of life (QoL). Therefore, this study aimed to identify the factors affecting the QoL and mental status of breast cancer patients with schizophrenia, thus enabling subsequent interventions to improve their mental status and QoL.

**Methods:**

This study included 125 breast cancer patients with schizophrenia, and their mental status and QoL were analyzed before and after surgery. The mental status of these patients was assessed using the Symptom Checklist-90 (SCL-90), and the World Health Organization Quality of Life-BREF (WHOQOL-BREF) was used to score their QoL. Baseline characteristics were recorded, including age, marital status, education level, and per capita monthly household income. The clinical and demographic data were statistically analyzed to identify factors affecting patients’ mental status and QoL.

**Results:**

We observed that the mental status and QoL of breast cancer patients with schizophrenia at preoperative and postoperative stages were influenced by marital status, education level, tumor stage, tumor size, and monthly family income. Additionally, the type of surgery was significantly associated with postoperative mental status and QoL and was found to be a predictor influencing the overall QoL. Furthermore, surgery had a positive impact on patients across different treatment stages.

**Conclusion:**

The psychological state and QoL of breast cancer patients with schizophrenia are influenced by various factors at different stages of treatment (preoperative and postoperative). Surgery significantly improves the patients’ psychological state and QoL.

## Introduction

As reported by the American Cancer Society in 2024, breast cancer (BC) accounts for 32.0% of the estimated new cases in women, with an annual incidence increase of 0.6%. Currently, BC has become the most prevalent malignancy among women [1]. Schizophrenia is a chronic mental disorder affecting approximately 1% of the global population. Compared to patients without schizophrenia, those with the disorder have a higher risk of mortality following cancer treatment and a shorter life expectancy [2]. Studies indicate that patients with schizophrenia exhibit abnormal mental states, highlighting the significance of addressing mental health concerns [3], such as the effect of stigma [4]. Their quality of life is also significantly impacted [5].

However, the correlation between preoperative and postoperative impacts on mental state and quality of life in breast cancer patients with schizophrenia remains inconclusive. The clinical symptoms of breast cancer often include nipple discharge, breast lumps, and areolar abnormalities, which can sometimes be mistaken for conditions such as mammary gland hyperplasia. Breast cancer is characterized by its invasiveness and potential for metastasis; if not treated promptly, it can damage other organs and increase mortality risk [6, 7, 8]. Surgery is the primary treatment for breast cancer, with the surgical approach selected based on the tumor stage [9, 10, 11]. Early-stage breast cancer is often treated with breast-conserving surgery [12, 13], while advanced stages typically require mastectomy [14]. Postoperative treatments usually include radiotherapy and chemotherapy to inhibit tumor proliferation and reduce the risk of recurrence [15, 16]. However, a breast cancer diagnosis imposes significant psychological stress on patients. Various physiological and psychological stressors during treatment, along with postoperative changes in breast appearance, can exacerbate this burden, substantially impacting patients’ quality of life (QoL). Emotional challenges such as depression and anxiety may develop after diagnosis or during treatment, leading to negative attitudes, reluctance to undergo treatment, or diminished confidence in its effectiveness, which can delay disease management [17]. Patients with mental health disorders may fail to adhere to medical advice, often neglecting treatment protocols by missing medication doses, refusing necessary treatments, or discontinuing their treatment plans altogether. In more severe cases, these patients may exhibit self-harming behaviors or suicidal tendencies, which threaten their safety and complicate the overall treatment of their condition [18].

The preoperative and postoperative phases are critical in breast cancer treatment, during which patients’ mental status and QoL are influenced by multiple factors [19, 20]. In the preoperative phase, patients often experience anxiety and fear about the diagnosis, while in the postoperative stage, they may experience surgical trauma, side effects from chemotherapy, and uncertainty about the future [21]. Understanding the factors affecting mental status and QoL at each treatment stage is crucial for developing personalized interventions to improve treatment outcomes and overall QoL.

Patients with breast cancer who also have schizophrenia may experience more severe challenges to their mental state and quality of life [22]. Breast cancer alone imposes significant stress on a patient’s physical and mental health, and the presence of schizophrenia further complicates their situation, increasing the overall impact.

This study aims to comprehensively evaluate the factors influencing the mental status and QoL of breast cancer patients with schizophrenia at different treatment stages (preoperative and postoperative). It will explore relevant physiological, psychological, and social factors to provide targeted clinical care approaches that improve their psychological state and enhance their QoL post-surgery.

## Materials and Methods

### General Information

Clinical data were collected from 125 breast cancer patients with schizophrenia who were treated at Shaoxing People’s Hospital between January 2023 and April 2024. The mental well-being and QoL of the study participants were analyzed preoperatively and postoperatively. Based on the median score of the Symptom Checklist-90 (SCL-90), patients were divided into two groups: the good mental status group and the poor mental status group [23, 24]. Moreover, based on the World Health Organization Quality of Life-BREF (WHOQOL-BREF) scores, patients were categorized into good QoL and poor QoL groups [25, 26]. This study was approved by the institutional ethics committee, and its design adhered to the principles of the Declaration of Helsinki. Furthermore, informed consent was obtained from all study participants.

We assessed patients’ mental state and quality of life one week before surgery and one month after surgery. This timeframe was selected based on clinical experience and relevant literature to capture the short-term and mid-term effects of the procedure. Strict quality control measures were implemented throughout the procedure. All assessments were conducted by trained independent evaluators to ensure consistency and objectivity. To avoid bias, a random sampling method was used to select patients, and double-blind assessments were utilized to ensure that evaluators were unaware of the specific patient details. Additionally, standardized and validated assessment tools, such as the SCL-90 and WHOQOL-BREF, were employed to ensure the reliability and validity of the data.

Baseline data were collected from the patients one week before surgery, including patients’ basic demographic information (like age, income, and education level), mental state, and quality of life. Structured questionnaires and clinical assessment forms were used to collect this data, ensuring that all data were obtained under uniform conditions to minimize the risk of systemic bias.

Inclusion criteria for the study participants were set as follows: histopathologically confirmed breast cancer patients aged ≥18 years, with normal cognitive function, and those with complete clinical data. Furthermore, BC patients who were concurrently diagnosed with schizophrenia by a clinical psychiatrist were also included in this study. Clinical Evaluation included a detailed medical and psychiatric history, including symptomatology, duration, and the impact on daily functioning. Patients were diagnosed following the Diagnostic and Statistical Manual of Mental Disorders, 5th Edition (DSM-5), with the presence of at least two of the following symptoms, each present for a significant portion of time during one month (or less if successfully treated), with at least one of the first three: Delusions, Hallucinations, Disorganized speech (e.g., frequent derailment or incoherence), Grossly disorganized or catatonic behavior, and Negative symptoms (e.g., diminished emotional expression or avolition) [27].

After the diagnosis of schizophrenia, breast cancer patients received appropriate pharmacological treatment to manage their symptoms of schizophrenia. Patients then underwent relevant radiotherapy and chemotherapy according to their surgical status and disease progression. As a result, 125 patients (serial numbers s001–s125) met the inclusion criteria.

Exclusion criteria included concurrent liver or kidney failure, poor cardiopulmonary function, presence of other malignancies, and recurrence of breast cancer.

### Clinical Data Collection

The mental status and overall QoL of breast cancer patients with schizophrenia at different treatment stages were collected and compared between the two experimental groups.

Mental status was assessed using the Symptom Checklist-90 (SCL-90), which includes 90 items across nine factors: somatization, obsessive-compulsive symptoms, interpersonal sensitivity, depression, anxiety, hostility, phobic anxiety, paranoid ideation, and psychoticism. Each item is scored on a scale of 1–5, with 1 indicating no symptoms and 5 indicating severe symptoms. The scoring criteria were as follows: (a) A total score exceeding 160 indicates positive symptoms. (b) More than 43 positive items (with a factor score ≥2) indicate potential issues. (c) Factor scores were interpreted as follows: <1.5 indicates no symptoms, 2.0–2.9 indicates mild symptoms, 3.0–3.8 indicates moderate symptoms, and >3.8 indicates severe symptoms. Based on their SCL-90 scores, they were divided into a good mental status group (90–159) and a poor mental status group (160–450) [23, 24].

QoL was evaluated using the WHOQOL-BREF, a self-assessment scale developed by the World Health Organization. This scale includes 26 items distributed across four domains: physical health, psychological health, social relationships, and environment. Each item is scored on a scale from 1 (very dissatisfied) to 5 (very satisfied), with a total score ranging from 26 to 130. Higher scores indicate better QoL. Scores ≥60% of the total (≥3 per item) are considered satisfactory, while scores <3 are unsatisfactory. Based on their scores, patients were divided into a poor QoL group (26–71) and a good QoL group (72–130) [28].

### Data Processing

Data were statistically analyzed using SPSS 22.0 (IBM, Corp., Armonk, NY, USA). The normality of the data was determined using the Shapiro-Wilk test. Since the data did not follow a normal distribution, the measurement data were presented as median (25th percentile, 75th percentile). Comparisons between groups were conducted using non-parametric tests, specifically the Wilcoxon signed-rank test. Categorical data were expressed as frequencies [n (%)] and analyzed using the chi-square test. A *p*-value < 0.05 was considered statistically significant.

## Results

### Comparison of Mental Status and QoL in Breast Cancer Patients With Schizophrenia at Preoperative and Postoperative Treatment Stages

This study assessed patients’ mental status and QoL before and after surgery. The results showed that mental status and QoL improved postoperatively compared to preoperative levels. As shown in Tables 1,2, postoperative SCL-90 scores, including somatization, interpersonal sensitivity, depression, and anxiety, were significantly lower than preoperative scores (*p* < 0.05). Additionally, the QoL of breast cancer patients with schizophrenia exhibited significant improvement after surgery, with higher scores found in physical, psychological, social, and environmental domains.

**Table 1.** **Comparison of mental status (SCL-90 scores) in breast cancer patients with schizophrenia at different treatment stages (preoperative and postoperative) (median (25th percentile, 75th percentile))**.

Experimental group	SCL-90									
	Somatization	Obsessive-compulsive	Interpersonal sensitivity	Depression	Anxiety	Hostility	Phobic anxiety	Paranoid ideation	Psychoticism	Total scores
Preoperative (n = 125)	31 (19.5, 36.5)	30 (14, 33)	17 (13, 20)	27 (22, 432)	18 (13, 24)	11 (10, 13)	19 (15, 23)	14 (13, 16)	20 (19, 24)	207 (149.5, 243.5)
Postoperative (n = 125)	28 (15, 33.5)	26 (12, 30)	14 (10, 17)	24 (15, 29)	13 (10, 19.5)	8 (7, 11)	17 (12.5, 21)	12 (10.5, 14)	19 (15, 20)	180 (124, 215.5)
Z-value	–7.82	–7.94	–7.07	–8.84	–9.33	–7.11	–7.51	–7.28	–8.73	–8.97
*p*-value	<0.05^*^	<0.05^*^	<0.05^*^	<0.05^*^	<0.05^*^	<0.05^*^	<0.05^*^	<0.05^*^	<0.05^*^	<0.05^*^

Note: ^*^p < 0.05, SCL-90, Symptom Checklist-90, comparisons between groups were conducted using the Wilcoxon signed-rank test.

**Table 2.** **Comparison of QoL (WHOQOL-BREF Scores) in breast cancer patients with schizophrenia at different treatment stages (preoperative and postoperative) (median (25th percentile, 75th percentile))**.

Group	QoL				
	Physical health	Psychological health	Social relationships	Environment	Total scores
Preoperative (n = 125)	19 (16, 21)	17 (15, 19)	21 (17, 25)	14 (11, 15.5)	73 (67, 83)
Postoperative (n = 125)	20 (18, 23)	18 (16, 20)	22 (19.5, 26)	15 (13, 17)	82 (73, 89)
Z-value	–5.13	–2.97	–4.68	–5.43	–9.64
*p*-value	<0.05^*^	<0.05^*^	<0.05^*^	<0.05^*^	<0.05^*^

Note: ^*^p < 0.05, QoL, quality of life, comparisons between groups were conducted using the Wilcoxon signed-rank test.

### Analysis of Factors Affecting Preoperative Mental Status in Breast Cancer Patients With Schizophrenia

Patients undergoing preoperative chemotherapy were treated with anthracycline drugs, such as epirubicin hydrochloride injection and doxorubicin hydrochloride injection, following medical advice. Preoperative radiotherapy was primarily used for patients with locally advanced breast cancer, aiming to convert some inoperable cases into operable ones. To evaluate the factors influencing the preoperative mental status of breast cancer patients with schizophrenia, a univariate analysis was conducted. Patients were divided into a good mental status group (SCL-90 total score 90–159, 39 patients) and a poor mental status group (SCL-90 total score 160–450, 86 patients). The results revealed that marital status, educational level, tumor stage, tumor size, and monthly household income (*p* < 0.05, Table 3) were significant factors affecting the preoperative mental status of breast cancer patients with schizophrenia.

**Table 3.** **Univariate analysis of factors affecting preoperative mental status in breast cancer patients with schizophrenia (n (%))**.

Subjects	Groups	Poor mental	Good mental	χ^2^	*p*-value
		n = 86 (%)	n = 39 (%)		
Age	30–50	27 (31.40)	11 (28.20)	3.36	0.19
	51–59	28 (32.56)	19 (48.72)		
	60–85	31 (36.04)	9 (23.08)		
Marital status	Divorced	50 (58.14)	9 (23.08)	13.89	<0.05^*^
	Single	14 (16.28)	9 (23.08)		
	Married	22 (25.58)	21 (53.85)		
Educational level	≤Middle school	36 (41.86)	8 (20.51)	9.63	<0.05^*^
	≥College	21 (24.42)	20 (51.28)		
	High school	29 (33.72)	11 (28.21)		
Tumor stage	Stage I	21 (24.42)	26 (66.67)	20.74	<0.05^*^
	Stage II	33 (38.37)	8 (20.51)		
	Stage III	32 (37.21)	5 (12.82)		
Tumor size	≤2 cm	28 (32.56)	24 (61.54)	11.78	<0.05^*^
	2 < x ≤ 5 cm	41 (47.67)	14 (35.90)		
	>5 cm	17 (19.77)	1 (2.56)		
Payment method	Insurance	44 (51.16)	25 (64.10)	1.82	0.18
	Self-pay	42 (48.84)	14 (35.90)		
Monthly household income	≤711 USD	49 (56.98)	5 (12.82)	21.32	<0.05^*^
	>711 USD	37 (43.02)	34 (87.18)		
Chemotherapy/radiotherapy	No	34 (39.53)	14 (35.90)	0.15	0.70
	Yes	52 (60.47)	25 (64.10)		

Note: ^*^p < 0.05, chi-square test.

The significant indicators from the univariate analysis (marital status, educational level, tumor stage, tumor size, monthly household income) were assigned values as shown in Table 4, and a binary logistic regression analysis was performed. The findings demonstrated that tumor stage and monthly household income were predictors (*p* < 0.05) affecting patients’ preoperative mental status, as detailed in Table 5.

**Table 4.** **Assignment of values for significant indicators in univariate analysis**.

Factors	Divisor	Assignment
Marital status	X1	Divorced = 1, Single = 2, Married = 3
Educational level	X2	≤Middle school = 1, ≥College = 2, High school = 3
Tumor stage	X3	Stage I = 1, Stage II = 2, Stage III = 3
Tumor size	X4	≤2 = 1, 2 < x ≤5 = 2, >5 = 3
Surgical method	X5	Breast-conserving surgery = 1, Mastectomy = 2
Payment method	X6	medical insurance = 1, Self-pay = 2
Monthly household income	X7	Monthly profit ≤711 USD = 1, Monthly profit >711 USD = 2
Chemotherapy/radiotherapy	X8	No = 1, Yes = 2
Mental status	X9	Score ≥160 = 1, Score <160 = 2
QoL level	X10	Score <72 = 1, Score ≥72 = 2

Note: QoL, quality of life.

**Table 5.** **Binary logistic regression analysis of factors affecting preoperative mental status**.

Factors	β	SE	Wald	*p*-value	OR (95% CI)
Marital status	0.44	0.28	2.47	0.12	1.56 (0.90∼2.70)
Educational level	–0.13	0.32	0.17	0.68	0.88 (0.47∼1.64)
Tumor stage	–1.06	0.49	4.66	0.03^*^	0.35 (0.13∼0.91)
Tumor size	0.09	0.55	0.03	0.87	1.10 (0.37∼3.24)
Monthly household income	1.90	0.57	11.24	<0.05^*^	6.70 (2.20∼20.36)

Note: ^*^p < 0.05, β, regression coefficient; SE, standard error; OR, odds ratio; CI, confidence interval.

### Analysis of Factors Affecting Postoperative Mental Status in Breast Cancer Patients With Schizophrenia

Postoperative chemotherapy was administered under medical guidance using drugs such as paclitaxel injection and doxorubicin hydrochloride injection. Postoperative radiotherapy is an adjuvant treatment following mastectomy aimed at eradicating potential residual lesions and preventing or reducing recurrence. To evaluate the factors influencing the mental status of breast cancer patients with schizophrenia post-surgery, a univariate analysis was conducted. Based on the SCL-90 total scores, patients were divided into a good mental status group (90–159, 45 patients) and a poor mental status group (160–450, 80 patients). As presented in Table 6, marital status, educational level, tumor stage, surgical method, and monthly household income were significant factors influencing the postoperative mental status of breast cancer patients with schizophrenia (*p* < 0.05).

**Table 6.** **Univariate analysis of factors affecting postoperative mental status in breast cancer patients with schizophrenia (n (%))**.

Subjects	Groups	Poor mental	Good mental	χ^2^	*p*-value
		n = 80 (%)	n = 45 (%)		
Age	30–50	24 (30.00)	14 (31.11)	3.65	0.1612
	51–59	26 (32.50)	21 (46.67)		
	60–85	30 (37.50)	10 (22.22)		
Marital status	Divorced	50 (62.50)	9 (20.00)	24.51	<0.05^*^
	Single	14 (17.50)	9 (20.00)		
	Married	16 (20.00)	27 (60.00)		
Educational level	≤Middle school	36 (45.00)	8 (17.78)	17.79	<0.05^*^
	≥College	16 (20.00)	25 (55.56)		
	High school	28 (35.00)	12 (26.67)		
Tumor stage	Stage I	18 (22.50)	29 (64.44)	22.16	<0.05^*^
	Stage II	31 (38.75)	10 (22.22)		
	Stage III	31 (38.75)	6 (13.33)		
Surgical method	Breast-conserving surgery	46 (57.50)	36 (80.00)	6.46	0.01^*^
	Mastectomy	34 (42.50)	9 (20.00)		
Payment method	Insurance	39 (48.75)	30 (66.67)	3.74	0.05
	Self-pay	41 (51.25)	15 (33.33)		
Monthly household income	≤711 USD	49 (61.25)	5 (11.11)	29.51	<0.05^*^
	>711 USD	31 (38.75)	40 (88.89)		
Chemotherapy/radiotherapy	No	32 (40.00)	16 (35.56)	0.24	0.62
	Yes	48 (60.00)	29 (64.44)		

Note: ^*^p < 0.05, chi-square test.

The variables were assigned values, as shown in Table 4, followed by a binary logistic regression analysis. Furthermore, our results indicated marital status, tumor stage, and monthly household income as predictors influencing postoperative mental status (*p* < 0.05), as detailed in Table 7.

**Table 7.** **Binary logistic regression analysis of factors affecting postoperative mental status**.

Factors	β	SE	Wald	*p*-value	OR (95% CI)
Marital status	0.84	0.31	7.48	0.01^*^	2.31 (1.27∼4.19)
Educational level	–0.40	0.35	1.31	0.25	0.67 (0.34∼1.33)
Tumor stage	–1.24	0.54	5.37	0.02^*^	0.29 (0.10∼0.83)
Surgical method	–0.10	0.56	3.18	0.08	0.37 (0.12∼1.10)
Monthly household income	2.38	0.61	15.11	<0.05^*^	10.79 (3.25∼35.79)

Note: ^*^p < 0.05, β, regression coefficient; SE, standard error; OR, odds ratio; CI, confidence interval.

### Analysis of Factors Affecting Preoperative QoL in Breast Cancer Patients With Schizophrenia

To evaluate the factors influencing the preoperative QoL in breast cancer patients with schizophrenia, a univariate analysis was performed. Based on the total QoL scores, patients were divided into a good QoL group (72–130 points, 73 patients) and a poor QoL group (26–71 points, 52 patients). As detailed in Table 8, marital status, educational level, tumor stage, tumor size, medical insurance coverage, monthly household income, and experience with chemotherapy/radiotherapy were significant factors affecting the preoperative QoL in breast cancer patients with schizophrenia (*p* < 0.05).

**Table 8.** **Univariate analysis of factors affecting preoperative QoL in breast cancer patients with schizophrenia**.

Subjects	Groups	Poor QoL group	Good QoL group	χ^2^	*p*-value
		n = 52 (%)	n = 73 (%)		
Age	30–50	15 (28.85)	23 (31.51)	1.80	0.41
	51–59	17 (32.69)	30 (41.10)		
	60–85	20 (38.46)	20 (27.40)		
Marital status	Divorced	39 (75.00)	20 (27.40)	39.11	<0.05^*^
	Single	11 (21.15)	12 (16.44)		
	Married	2 (3.85)	41 (56.16)		
Educational level	≤Middle school	35 (67.31)	9 (12.33)	40.77	<0.05^*^
	≥College	7 (13.46)	34 (46.58)		
	High school	10 (19.23)	30 (41.10)		
Tumor stage	Stage I	11 (21.15)	36 (49.32)	25.74	<0.05^*^
	Stage II	13 (25.00)	28 (38.36)		
	Stage III	28 (53.85)	9 (12.33)		
Tumor size	≤2 cm	14 (26.92)	38 (52.54)	11.89	0.0126^*^
	2 < x ≤ 5 cm	25 (48.08)	30 (41.10)		
	>5 cm	13 (25.00)	5 (6.85)		
Payment method	Insurance	22 (42.31)	47 (64.38)	5.99	0.01^*^
	Self-pay	30 (57.69)	26 (35.62)		
Monthly household income	≤711 USD	36 (69.23)	18 (24.66)	24.59	<0.05^*^
	>711 USD	16 (30.77)	55 (75.34)		
Chemotherapy/radiotherapy	No	27 (51.92)	21 (28.77)	6.88	0.01^*^
	Yes	25 (48.08)	52 (71.23)		

Note: ^*^p < 0.05, QoL, quality of life, chi-square test.

The variables were assigned values (Table 4), followed by a binary logistic regression analysis. The findings demonstrated that marital status, educational level, tumor stage, monthly household income, and experience with chemotherapy/radiotherapy were predictors influencing preoperative QoL (*p* < 0.05), as detailed in Table 9.

**Table 9.** **Binary logistic regression analysis of factors affecting preoperative QoL**.

Factors	β	SE	Wald	*p*-value	OR (95% CI)
Marital status	1.24	0.39	9.93	0.01^*^	3.45 (1.60∼7.46)
Educational level	1.33	0.37	12.86	<0.05^*^	3.80 (1.83∼7.87)
Tumor stage	–1.04	0.50	4.35	0.04^*^	0.35 (0.13∼0.94)
Tumor size	0.13	0.56	0.05	0.82	1.14 (0.38∼3.44)
Payment method	–0.23	0.59	0.15	0.70	0.80 (0.25∼2.51)
Monthly household income	1.63	0.58	7.84	0.01^*^	5.11 (1.63∼16.01)
Chemotherapy/radiotherapy	1.51	0.62	6.05	0.01^*^	4.55 (1.36∼15.20)

Note: ^*^p < 0.05, β, regression coefficient; SE, standard error; OR, odds ratio; CI, confidence interval; QoL, quality of life.

### Analysis of Predictors Affecting Postoperative QoL in Breast Cancer Patients With Schizophrenia

A univariate analysis was conducted to identify the predictors influencing the postoperative QoL in breast cancer patients with Schizophrenia. Based on the total QoL scores, patients were divided into a good QoL group (72–130 points, 104 patients) and a poor QoL group (26–71 points, 21 patients). As shown in Table 10, we observed that marital status, educational level, tumor stage, surgical method, payment method, monthly household income, and experience with chemotherapy/radiotherapy substantially affected postoperative QoL in breast cancer patients (*p* < 0.05).

**Table 10.** **Univariate analysis of factors affecting postoperative QoL in breast cancer patients with schizophrenia**.

Subjects	Groups	Poor QoL group	Good QoL group	χ^2^	*p*-value
		n = 21 (%)	n = 104 (%)		
Age	30–50	6 (28.57)	32 (30.77)	1.51	0.47
	51–59	6 (28.57)	41 (39.42)		
	60–85	9 (42.86)	31 (29.81)		
Marital status	Divorced	18 (85.71)	41 (39.42)	15.02	<0.05^*^
	Single	1 (4.76)	22 (21.15)		
	Married	2 (9.52)	41 (39.42)		
Educational level	≤Middle school	15 (71.43)	29 (27.88)	14.85	<0.05^*^
	≥College	4 (19.05)	37 (35.58)		
	High school	2 (9.52)	38 (36.54)		
Tumor stage	Stage I	5 (23.81)	42 (40.38)	12.81	<0.05^*^
	Stage II	3 (14.29)	38 (36.54)		
	Stage III	13 (61.90)	24 (23.08)		
Surgical method	Breast-conserving surgery	6 (28.57)	76 (73.08)	15.34	<0.05^*^
	Mastectomy	15 (71.43)	28 (26.92)		
Payment method	Insurance	6 (28.57)	63 (60.58)	7.24	<0.05^*^
	Self-pay	15 (71.43)	41 (39.42)		
Monthly household income	≤711 USD	15 (71.43)	39 (37.50)	8.20	<0.05^*^
	>711 USD	6 (28.57)	65 (62.50)		
Chemotherapy/radiotherapy	No	14 (71.43)	34 (37.50)	8.53	<0.05^*^
	Yes	7 (28.57)	70 (62.50)		

Note: ^*^p < 0.05, QoL, quality of life, chi-square test.

The variables were assigned values, as shown in Table 4, followed by a binary logistic regression analysis. The results indicated that the surgical method and experience with chemotherapy/radiotherapy were predictors influencing postoperative QoL (*p* < 0.05), as detailed in Table 11.

**Table 11.** **Binary logistic regression analysis of predictors affecting postoperative QoL**.

Factors	β	SE	Wald	*p*-value	OR (95% CI)
Marital status	0.84	0.52	2.59	0.11	2.321 (0.832∼6.477)
Educational level	0.80	0.41	3.79	0.05	2.234 (0.994∼5.019)
Tumor stage	–0.42	0.38	1.17	0.28	0.66 (0.311∼1.402)
Surgical method	–1.83	0.66	7.73	0.01^*^	0.161 (0.044∼0.583)
Payment method	–1.08	0.70	2.41	0.12	0.34 (0.087∼1.329)
Monthly household income	0.85	0.69	1.51	0.22	2.335 (0.604∼9.026)
Chemotherapy/radiotherapy	1.50	0.65	5.36	0.02^*^	4.458 (1.258∼15.801)

Note: ^*^p < 0.05, β, regression coefficient; SE, standard error; OR, odds ratio; CI, confidence interval; QoL, quality of life.

## Discussion

The novelty and clinical significance of this study include the comprehensive analysis of multiple factors, the independent impact of surgery type, and dynamic observation across treatment stages. This study is the first to comprehensively analyze the mental status and QoL of breast cancer patients with schizophrenia, considering various socioeconomic and oncological variables. It underscores the role of these factors at different treatment stages, addressing a critical gap in the current research. Furthermore, this study identifies surgery type as a predictor significantly influencing overall QoL. These findings highlight the significance of carefully selecting surgical options during treatment planning to optimize patient outcomes. Additionally, by comparing preoperative and postoperative phases, our finding reveals the lasting positive effects of surgery on mental status and QoL, providing evidence-based guidance for rehabilitation and psychological interventions. This study offers novel insights into the clinical management and treatment strategies of breast cancer patients with schizophrenia, particularly in the context of personalized care and multidisciplinary collaboration.

Breast cancer alone already imposes significant stress and challenges on a patient’s physical and mental health, and the co-occurrence of schizophrenia further complicates this situation [29]. This dual diagnosis can lead to several challenges, including poor treatment adherence. Impaired cognitive function or emotional disturbances associated with schizophrenia may hinder patients from adhering to breast cancer treatment plans, resulting in suboptimal treatment outcomes [30]. Furthermore, these patients commonly experience increased psychological stress, decline in quality of life, lack of social support, and drug interaction [31]. However, the medications used to treat breast cancer and schizophrenia may interact, increasing the risk of side effects and complicating the treatment process [32].

Surgery and chemotherapy are primary treatments for breast cancer, but they cause varying levels of trauma [33]. Preoperatively, patients often face fear and anxiety, while postoperatively, they may experience pain, fatigue, and other physical discomforts [34, 35]. These physiological and psychological discomforts impact patients’ mental states and QoL [36, 37]. Effective pain management, postoperative care, and control of side effects are crucial in improving patients’ mental state and QoL [38].

This study assessed the mental state and QoL of breast cancer patients with schizophrenia at both preoperative and postoperative stages. The findings revealed a postoperative improvement in both mental state and QoL compared to preoperative levels. Specifically, postoperative SCL-90 scores for somatization, interpersonal sensitivity, depression, and anxiety significantly reduced, while QoL scores in the physical, psychological, social, and environmental domains significantly improved. These results are consistent with existing research and highlight multiple factors contributing to these changes [39, 40, 41].

The mental state of breast cancer patients with schizophrenia is crucial throughout the clinical treatment process. Maintaining a positive mental state and optimistic attitude can substantially improve treatment outcomes [19, 42]. Studies have shown that the mental state of these patients is often influenced by a complex interaction of factors, such as family support, personal understanding of the disease, and economic conditions [43, 44, 45]. These impacts can be attributed to several aspects, such as the preoperative and postoperative phases. In the preoperative phase, patients generally exhibit high levels of anxiety and depression, primarily associated with the uncertainty of their disease and fear of surgery [46]. Moreover, during this phase, the educational level of the patients affects their understanding of the disease. Better knowledge of the disease can help them understand their condition better, foster a positive mental outlook, and enhance treatment outcomes from diagnosis to surgery [47]. Our study revealed that marital status, educational level, tumor stage, tumor size, and household income are factors influencing the preoperative mental state of breast cancer patients with schizophrenia. Among these, tumor stage and household income were identified as predictor substantially influencing the preoperative mental state.

Furthermore, in the postoperative phase, the type of surgery affects the mental state of the patients. Changes in physical appearance, concerns about the future, and the psychological stress associated with long-term treatment may lead to depressive symptoms [48]. A well-organized recovery process post-surgery, including comprehensive rehabilitation plans, helps patients gradually adapt to the reality of their disease. Psychological support and counseling are critical in promoting emotional stability, while effective postoperative care alleviates physical discomfort, reducing the mental burden on patients [49].

The study findings indicate that marital status, tumor stage, and household income are predictors affecting the postoperative mental state of patients. Successful postoperative rehabilitation requires good marital status, family support, and sufficient financial conditions to provide psychological guidance and care. These results are consistent with previous research, which has reported that support from family, friends, and healthcare professionals can effectively reduce psychological stress in both preoperative and postoperative phases, enhancing patients’ confidence in overcoming the disease [50].

In addition to mental state, the QoL of breast cancer patients with schizophrenia has been extensively studied [51, 52, 53]. Support from family, friends, and healthcare teams is particularly crucial in the postoperative phase, providing strong emotional support. Social support is a vital factor influencing the QoL of breast cancer patients with schizophrenia [54]. Additionally, economic burden is a significant concern, especially during long-term treatment, as financial stress can further exacerbate psychological distress. Social and governmental policies, along with aid measures for breast cancer patients with schizophrenia, help alleviate their economic burden and improve their QoL [55].

This study demonstrated that marital status, educational level, tumor stage, tumor size, household income, insurance coverage, and chemotherapy/radiotherapy affected preoperative QoL. Among these factors, marital status, educational level, tumor stage, household income, and chemotherapy/radiotherapy were identified as predictors affecting preoperative QoL. In contrast, tumor size and insurance coverage were not observed as predictors.

For postoperative patients, the surgical method substantially impacted their mental state and QoL. Postoperatively, tumor size no longer affected the QoL. Instead, surgical methods and whether patients underwent chemotherapy/radiotherapy were identified as predictors affecting postoperative QoL. In summary, the results of this study suggest that married patients generally receive more family support, improving their mental health and QoL. Patients with higher education levels typically have better disease awareness and stronger self-adjustment abilities, positively impacting their mental state and QoL. Early-stage and smaller tumor patients usually have better prognoses, leading to higher postoperative recovery and QoL.

Compared to mastectomy, breast-conserving surgery causes less impact on the patient’s physical appearance and mental well-being, resulting in a higher postoperative QoL. Patients with better economic status can access superior medical resources and life support, positively affecting their recovery and QoL. Although chemotherapy/radiotherapy can cause side effects and physical discomfort, positive treatment outcomes can enhance their psychological confidence. Previous research findings are consistent with the results of this study, indicating improvement in the mental state and QoL of breast cancer with schizophrenia post-surgery. Several studies highlight that postoperative rehabilitation plans and psychological support significantly impact patients’ QoL. For instance, one study reported that comprehensive postoperative rehabilitation interventions significantly improved the physical and mental health of these patients. Additionally, multiple studies indicated the role of family and social support in facilitating postoperative recovery [56, 57].

Tailoring interventions to the specific characteristics of breast cancer patients with schizophrenia at different treatment stages is crucial for improving their QoL. Preoperatively, psychological counseling and education about the surgical process can help alleviate anxiety and fear, boosting patients’ confidence in the surgery. Postoperatively, comprehensive rehabilitation plans, including physical rehabilitation, psychological support, and social assistance, can improve faster recovery and enhance the overall QoL for patients. However, the limitations of this study include the need for a larger sample size to validate the generalizability of the findings regarding the effect of surgical procedures on the overall quality of life and mental status of these patients. Additionally, the study lacks long-term follow-up, which limits the ability to assess the lasting effects of surgery on mental status and QoL. Extended follow-up is needed for comparative results. Despite these limitations, this study presents significant innovation by examining the mental status and QoL of breast cancer patients with schizophrenia, an area that is often underexplored. This approach comprehensively explains how comorbid psychiatric conditions impact treatment outcomes and patient well-being. Through multifactorial analysis, factors such as age, marital status, education level, and income are considered, providing detailed insights into how these demographic variables interact with the effects of treatment on mental status and QoL. By identifying specific factors affecting mental status and QoL, this study may lead to the development of more targeted strategies to support breast cancer patients with schizophrenia, thereby improving their overall treatment experience.

## Conclusion

In summary, the mental state and QoL of breast cancer patients with schizophrenia at both preoperative and postoperative stages are influenced by various factors. Comprehensive evaluation and intervention targeting these factors can effectively improve the mental state and QoL of patients, ensuring better overall treatment outcomes. Future studies should focus on the effects of personalized interventions and develop more comprehensive support systems to help patients manage the challenges of the disease and enhance their QoL.

## Availability of Data and Materials

The data used and/or analyzed during the current study are available from the corresponding author upon reasonable request.
